# Supplementary material for: Sulfate limitation increases specific plasmid DNA yield and productivity in E. coli fed-batch processes
Source: Microb Cell Fact. 2023 Nov 28;22:242. doi: 10.1186/s12934-023-02248-2 (PMC10685491; doi:10.1186/s12934-023-02248-2)
Supplement: Supplementary file 1 — Additional file 1: Table S1. Parameters for dFBA simulations. Cells marked with "–" are not applicable and cells marked with "var" are not constant in the respective process. Table S2. Growth media components of the experiments. Growth media were sterilized by filtration. The trace element solution was prepared in 5 M HCl and contained (g/L): 4.41 CaCl2 ⋅ 2H2O, 3.34 FeSO4 ⋅ 7H2O, 1.43 CoCl2 ⋅ 6H2O, 1.03MnSO4 ⋅H2O, 0.15 CuSO4 ⋅ 5H2O, 0.17 ZnSO4 ⋅ 7H2O. † In total, 9.6 mL of a 200 g/L MgSO4 ⋅ 7 H2O stock solution were pulsed into the bioreactor (3.2 mL at feed start, 3.2 mL at 7 h after feed start and 3.2 mL at 15 h after feed start). ‡ According to our stoichiometric model [29], the amount of MgSO4 ⋅ 7 H2O in the batch medium alone can support more than 50 g of biomass. However, to ensure that Mg2+ is present in excess throughout the sulfate limited process we further added Mg2+ in the form of MgCl2 ⋅ 6 H2O. Table S3. Average values of several process variables of interest and their p-values compared to the control at 31 h (one-sided t-test). ccc pDNA corresponds to the supercoiled fraction of plasmid DNA measured.Table S4. List of potential targets for process optimization with sulfate limitation. We used lexicographic FBA (Table 1) to calculate maximal theoretical production rates during sulfate starvation (max. q∗) and sulfate excess (max. qμ) in mmol g−1 h−1. Compounds marked with † are displayed with their BiGG ID [75] to shorten the name. Previously described sulfate limitation target mevalonate is not naturally synthesized in E. coli [43] and, thus, not present in the list. Figure S1. Predicted optimal timing in a ${\text {SO}_4^{2-}}$SO42- limited linear fed-batch (A, B, C) and batch (D, E, F) process. Figure S2. Predic [file 12934_2023_2248_MOESM1_ESM.pdf]

Additional file 1

A.1. Additional Tables

Parameter	Batch	Fed-Batch Exponential	Fed-Batch Linear	Unit
$C_{biomass}(0)$	0.02	3.04	3.04	$g\,L^{-1}$
$C_{glucose}(0)$	20	0	0	$g\,L^{-1}$
$C_{SO_4^{2-}}(0)$	0 ... 0.29	0 ... 3.8	0 ... 2.9	$g\,L^{-1}$
$C_{pDNA}(0)$	0	0	0	$g\,L^{-1}$
$C^{feed}_{glucose}$	–	330	330	$g\,L^{-1}$
$V(0)$	0.5	0.5	0.5	L
$V(t_{end})$	0.5	1	1	L
t_{end}	var	35.2	36.0	h
k_M	2.7 [73]	–	–	$mg\,L^{-1}$
μ	var	0.1	var	h^{-1}
$q^{max}_{glucose}$	1.9 [74]	–	–	$g\,g^{-1}\,h^{-1}$
q^{μ}_{pDNA}	4.9	4.9	4.9	$mg\,g^{-1}\,L^{-1}$
q_{ATPM}	6.86	6.86	6.86	$mmol\,g^{-1}\,h^{-1}$
r^{feed}_i	–	Eq. (4b)	13.9	$mL\,h^{-1}$
$Y_{feed/biomass}$	–	10	–	$mL\,g^{-1}$

Table S1: **Parameters for dFBA simulations.** Cells marked with "–" are not applicable and cells marked with "var" are not constant in the respective process.

Component	Batch	Fed-Batch SO_4^{2-} limited	Fed-Batch control	Unit
glucose $\cdot$ H_2O	10.0	339.9		g/L
$Na_2HPO_4 \cdot 2\,H_2O$	7.38	7.38		g/L
KH_2PO_4	5.44	5.44		g/L
citric acid	2.0	2.0		g/L
NH_4Cl	2.0	0		g/L
$MgSO_4 \cdot 7\,H_2O$	0.24	3.8†	7.2	g/L
L-proline	0.20	6.0		g/L
L-isoleucie	0.20	6.0		g/L
bacto yeast extract	0.1	0		g/L
$MgCl_4 \cdot 6\,H_2O$	0	0.42‡	0	g/L
trace element solution	16.68	50		mL/L
thiamine-HCl (1%)	0.10	3.0		mL/L

Table S2: Growth media components of the experiments. Growth media were sterilized by filtration. The trace element solution was prepared in 5M HCl and contained (g/L): 4.41 $CaCl_2 \cdot 2H_2O$, 3.34 $FeSO_4 \cdot 7H_2O$, 1.43 $CoCl_2 \cdot 6H_2O$, 1.03 $MnSO_4 \cdot H_2O$, 0.15 $CuSO_4 \cdot$ 5H2O, 0.17 $ZnSO_4 \cdot 7H_2O$. † In total, 9.6 mL of a 200 g/L $MgSO_4 \cdot 7\,H_2O$ stock solution were pulsed into the bioreactor (3.2 mL at feed start, 3.2 mL at 7 h after feed start and 3.2 mL at 15 h after feed start). ‡ According to our stoichiometric model [29], the amount of $MgSO_4 \cdot 7\,H_2O$ in the batch medium alone can support more than 50 g of biomass. However, to ensure that Mg^{2+} is present in excess throughout the sulfate limited process we further added Mg^{2+} in the form of $MgCl_2 \cdot 6\,H_2O$.

Process Variable	Unit	CTRL	SLIM	Change	p-Value
C_{biomass}	g/L	44.233	37.750	−15%	1.0000
ccc fraction		0.878	0.904	+ 3%	0.0019**
$M_{\text{ccc pDNA}}$	g	2.060	2.316	+12%	0.0061**
$C_{\text{ccc pDNA}}$	g/L	2.220	2.519	+13%	0.0052**
$p_{\text{ccc pDNA}}$	g/L/h	0.072	0.081	+13%	0.0052**
$Y_{\text{ccc pDNA/biomass}}$	g/g	0.050	0.067	+33%	0.0001***
M_{pDNA}	g	2.346	2.564	+ 9%	0.0291*
C_{pDNA}	g/L	2.528	2.788	+10%	0.0243*
p_{pDNA}	g/L/h	0.082	0.090	+10%	0.0243*
$Y_{\text{pDNA/biomass}}$	g/g	0.057	0.074	+29%	0.0005***

Table S3: Average values of several process variables of interest and their p-values compared to the control at 31 h (one-sided t-test). ccc pDNA corresponds to the supercoiled fraction of plasmid DNA measured.

Compound	max. q^{μ}	max. q^{*}	Compound	max. q^{μ}	max. q^{*}
mththf†	0.000	9.610	(R)-Glycerate	0.000	21.367
(R)-Propane-1,2-diol	0.000	13.978	(S)-Propane-1,2-diol	0.000	13.839
ca4colipa†	0.000	0.183	1,5-Diaminopentane	0.000	7.723
2-Oxoglutarate	0.000	12.762	3-Hydroxypropanoate	0.000	11.744
4-Aminobutanoate	0.000	11.913	4-aminobenzoyl-glutamate	0.000	4.533
5-Dehydro-D-gluconate	0.000	10.529	Acetaldehyde	0.000	23.501
Acetate	0.000	28.795	Adenine	0.000	10.334
Adenosine	0.000	5.330	Agmatine	0.000	8.340
Allantoin	0.000	13.197	Citrate	0.000	11.530
Cytidine	0.000	6.131	D-Alanine	0.000	13.515
D-Alanyl-D-alanine	0.000	6.758	D-Gluconate	0.000	10.077
D-Glyceraldehyde	0.000	17.742	D-Lactate	0.000	20.000
Dihydroxyacetone	0.000	18.511	Enterochelin	0.000	1.704
Ethanol	0.000	20.000	Ethanolamine	0.000	10.830
fad†	0.000	1.769	FMN C17H19N4O9P	0.000	2.700
Fe(III)dicitrate	0.000	5.765	Fe-enterobactin	0.000	1.704
Formate	0.000	102.256	Fumarate	0.000	18.482
Glycerol 3-phosphate	0.000	9.606	Glycerol	0.000	15.637
Glycerophosphoglycerol	0.000	5.675	Glycine	0.000	27.012
Glycolate C2H3O3	0.000	28.795	Guanine	0.000	10.708
Guanosine	0.000	5.395	Hexanoate (n-C6:0)	0.000	7.500
Hypoxanthine	0.000	10.956	Indole	0.000	5.412
Inosine	0.000	5.459	L-Alanine	0.000	19.860
L-Arginine	0.000	8.274	L-Asparagine	0.000	15.935
L-Aspartate	0.000	17.975	L-Glutamate	0.000	11.815
L-Histidine	0.000	8.063	L-Homoserine	0.000	13.002
L-Idonate	0.000	10.077	L-Isoleucine	0.000	7.240
L-Lactate	0.000	17.376	L-Leucine	0.000	7.514
L-Lysine	0.000	7.723	L-Malate	0.000	18.482
L-Phenylalanine	0.000	5.428	L-Proline	0.000	10.019
L-Serine	0.000	20.626	L-Threonine	0.000	12.325
L-Tryptophan	0.000	4.465	L-Tyrosine	0.000	5.635
L-Valine	0.000	9.930	LalaDgluMdap†	0.000	3.174
LalaDgluMdapDala†	0.000	2.739	anhgm†	0.000	2.669
O-Acetyl-L-serine	0.000	11.800	Ornithine	0.000	9.478
nlipa†	0.000	0.397	Protoheme C34H30FeN4O4	0.000	1.301
Putrescine	0.000	9.565	Pyruvate	0.000	23.057
g3p†	0.000	6.361	Succinate	0.000	17.137
Thymidine C10H14N2O5	0.000	5.053	Thymine C5H6N2O2	0.000	10.311
Uracil	0.000	15.672	Urea CH4N2O	0.000	28.228
Uridine	0.000	6.510	Xanthine	0.000	11.921
Xanthosine	0.000	5.758			

Table S4: List of potential targets for process optimization with sulfate limitation. We used lexicographic FBA (Table 1) to calculate maximal theoretical production rates during sulfate starvation (max. q^{*}) and sulfate excess (max. q^{μ}) in mmol g^{-1} h^{-1}. Compounds marked with † are displayed with their BiGG ID [75] to shorten the name. Previously described sulfate limitation target mevalonate is not naturally synthesized in *E. coli* [43] and, thus, not present in the list.

A.2. Additional Figures

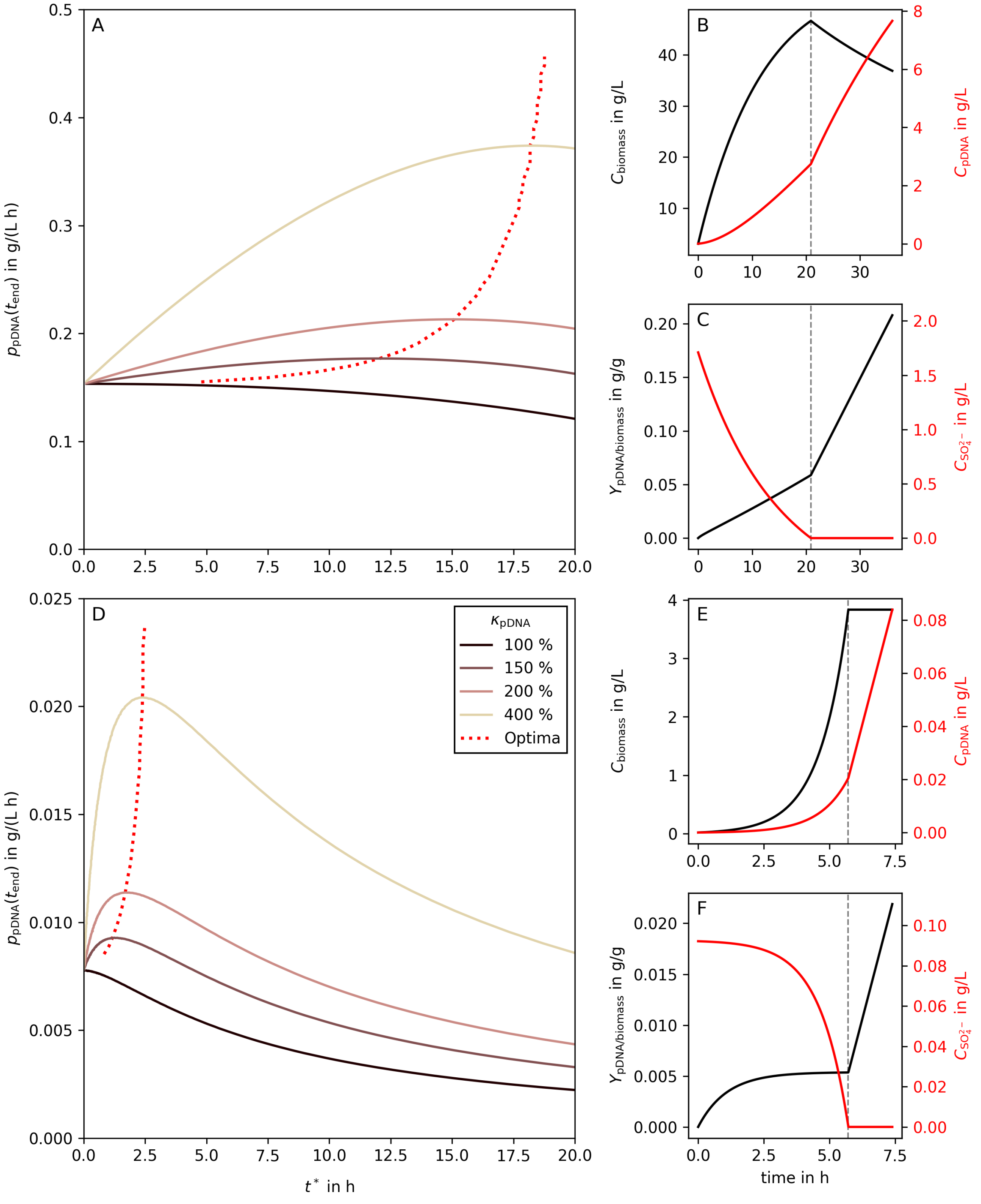

Figure S1: **Predicted optimal timing in a SO_4^{2-} limited linear fed-batch (panels A, B, C) and batch (D, E, F) process.** Panels **A** and **D** show average volumetric productivities of pDNA production ($\mathrm{g\,L^{-1}\,h^{-1}}$) as a function of the length of SO_4^{2-} starvation in the respective process (t^*). The full lines represent different levels of increased pDNA production during starvation as percentages of pDNA production rate during biomass growth (κ^*_{pDNA}). The dotted line indicates the location of the optima for κ^*_{pDNA} between 0 and 500 %. Panels **B**, **C** and **E**, **F** show the process curves of metabolites of interest in the optimal respective process for $\kappa^*_{\mathrm{pDNA}} = 200\,\%$. The gray dashed lines indicate the switching time point between the growth and production phases.

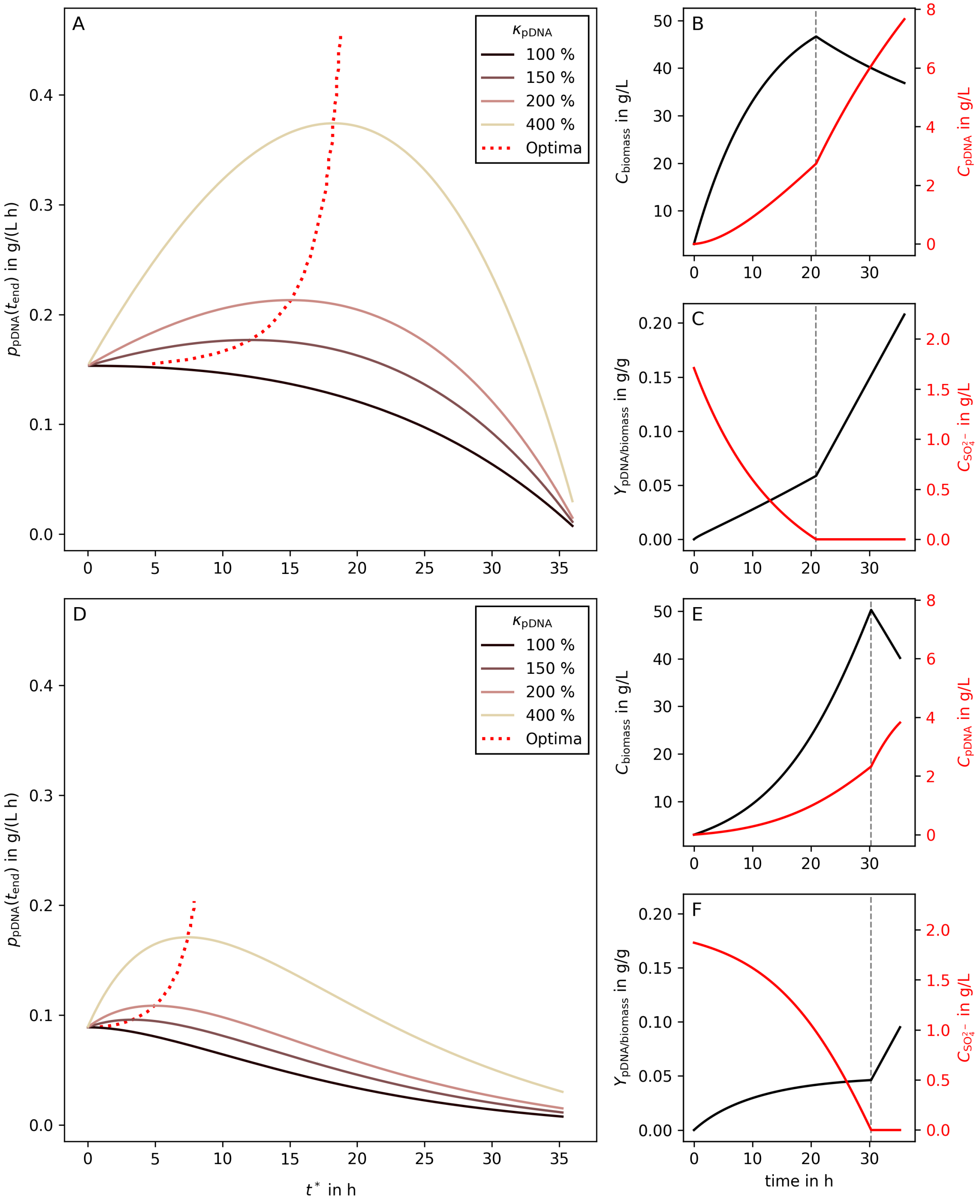

Figure S2: **Predicted optimal timing in a SO_4^{2-} limited linear (panels A, B, C) and exponential (D, E, F) fed-batch process.** Panels **A** and **D** show average volumetric productivities of pDNA production (g L^{-1} h^{-1}) as a function of the length of SO_4^{2-} starvation in the respective fed-batch process (t^*). The full lines represent different levels of increased pDNA production during starvation as percentages of pDNA production rate during biomass growth (κ^*_{pDNA}). The dotted line indicates the location of the optima for κ^*_{pDNA} between 0 and 500 %. Panels **B**, **C** and **E**, **F** show the process curves of metabolites of interest in the optimal respective process for κ^*_{pDNA} = 200 %. The gray dashed lines indicate the switching time point between the growth and production phases.

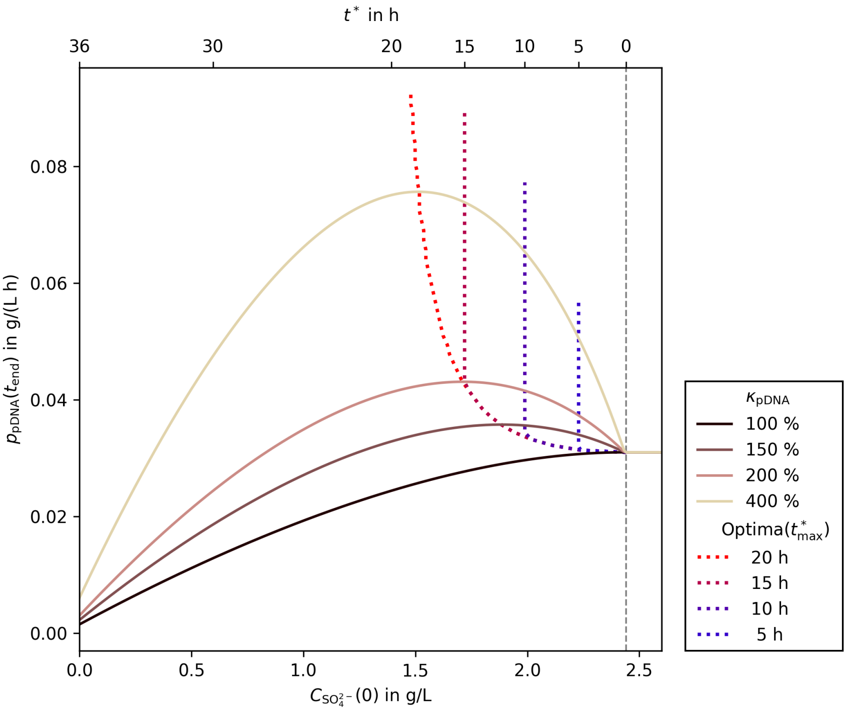

Figure S3: **Predicted optimal timing in a SO_4^{2-} limited linear fed-batch process with different maximum starvation lengths.** For models right of the gray dashed line no SO_4^{2-} limitation occurs. The colored dotted lines illustrate the optima of different maximum starvation phase lengths. The colored full lines illustrate different maximal pDNA production rates in the starvation phase.

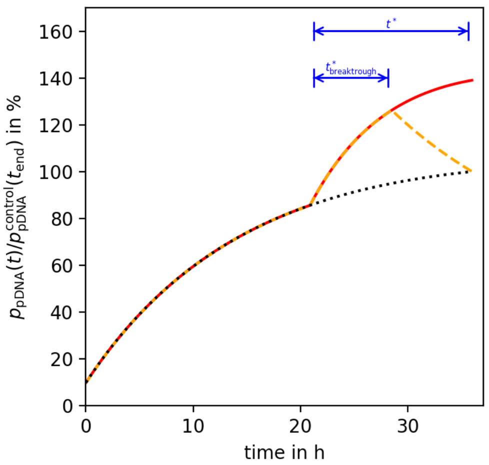

Figure S4: **Visualization explanation of $t^*_{breakthrough}$.** The black dotted line represents a non SO_4^{2-} limited control process. The processes represented by the red full and the orange dashed line start their SO_4^{2-} starvation at the optimal time point as calculated in Figure 4 ($\kappa_{pDNA} = 200\,\%$). The difference between them is that for the red full line, the pDNA production is held constant throughout the whole length of the starvation (t^*). In contrast, the orange dashed line describes p_{pDNA} when pDNA production occurs only in the first $t^*_{breakthrough}$ hours of starvation and is zero thereafter. $t^*_{breakthrough}$ describes the minimal length of pDNA production during starvation that leads to equal or better final productivities ($p_{pDNA}(t_{end})$) than the control.

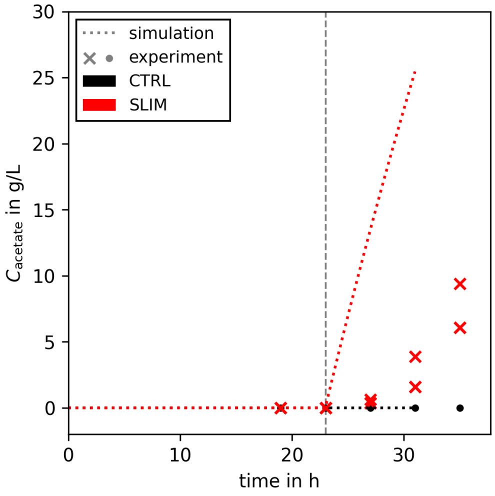

Figure S5: **Acetate accumulation during control and SO_4^{2-} limited processes.** The concentration of acetate over time was measured for one and two replicate(s) of CTRL and SLIM, respectively. Glucose concentrations were measured at the same time points and stayed at 0 g L^{-1} throughout the process (data not shown). Additionally, we performed dFBA simulations with time dependent q_{pDNA} (dotted lines, Figure 7B) and added the maximization of acetate flux at the end of the lexicographic objectives (Table 1). Acetate concentration according to the simulations are visualized as dotted lines.

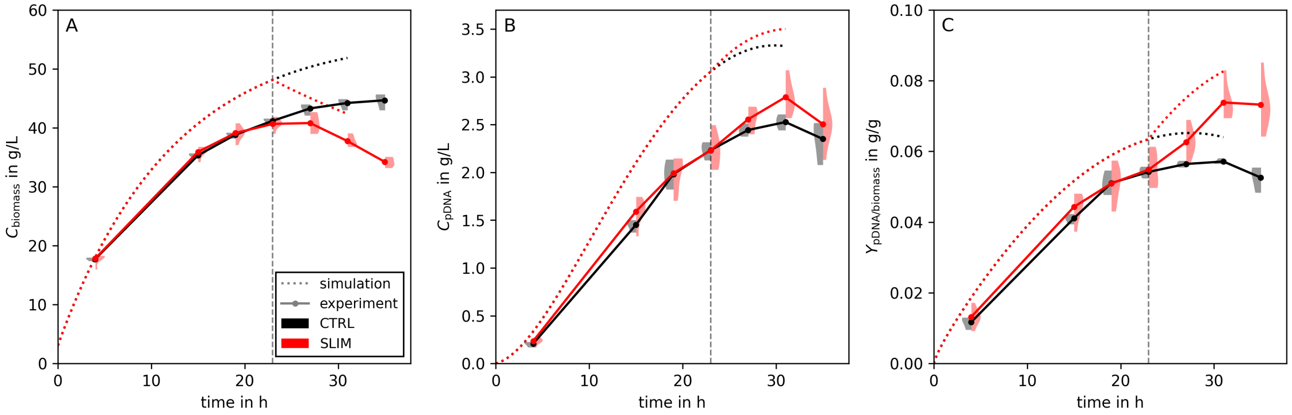

Figure S6: **Control and sulfate limited process with time-dependent q_{pDNA}.** A time-dependent, linearly declining $q_{pDNA}^{CTRL}(t)$ rate (black dotted line, Figure 7B) was fitted to the experimental data (black circles, Figure 7B). The rate of $q_{pDNA}^{SLIM}(t)$ was shifted in parallel to minimize the error of the flux ration at q_{pDNA} starved experimental data points (i.e., at t = 27 and 31 h, blue dotted line, Figure 7C). Subsequently, we performed dFBA. Results for biomass and pDNA concentration, and specific yield are plotted in panels A, B, and C, respectively. The simulation highlights that the assumption of constant q_{pDNA} is not necessary for process improvements by SO_4^{2-} limitation.

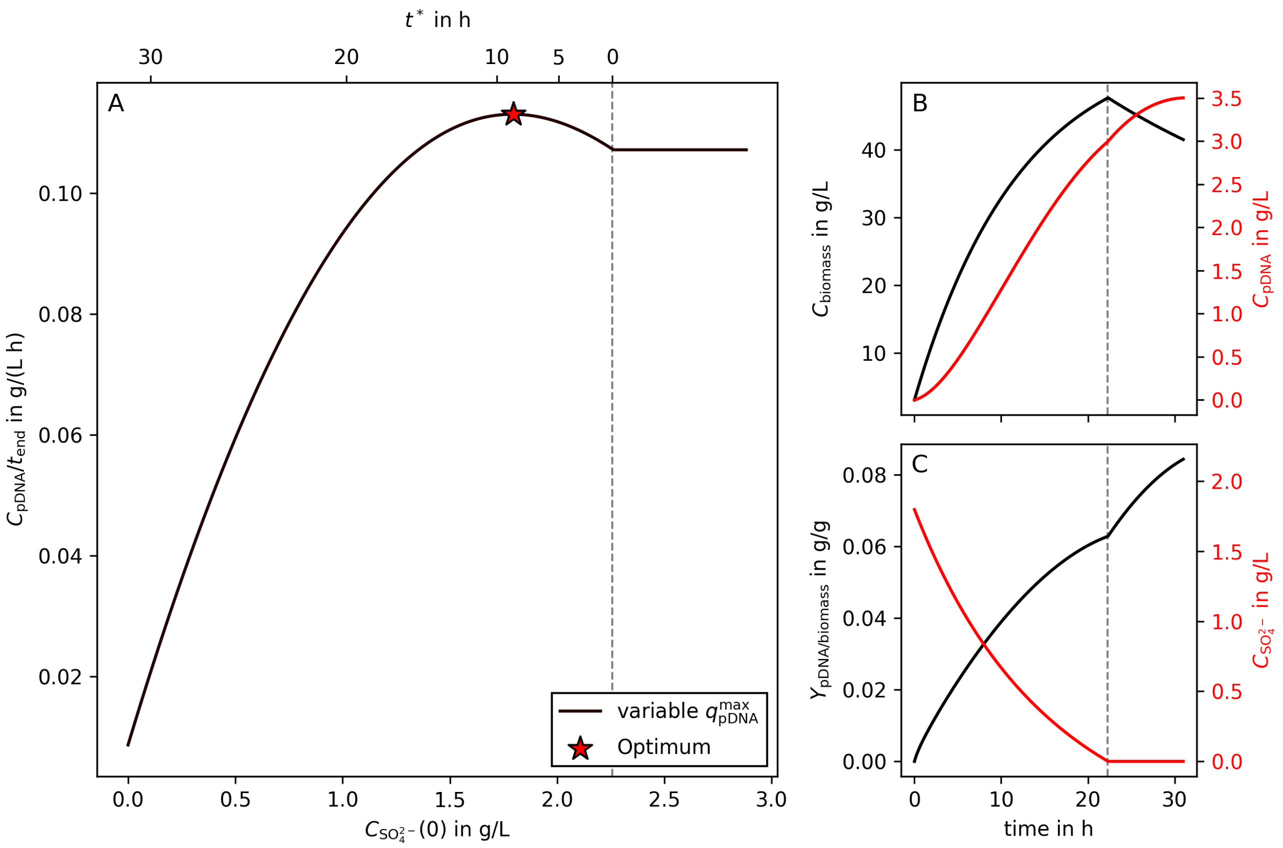

Figure S7: **Predicted optimal timing in a SO_4^{2-} limited linear fed-batch process with variable q_{pDNA}.** q_{pDNA} rates were fitted to CTRL and SLIM experimental values retroactively (Figure 7C). Panel **A** shows average volumetric productivities of pDNA production ($g\,L^{-1}\,h^{-1}$) as a function of the initial SO_4^{2-} concentration. The second X-axis of panel **A** (top) illustrates the length of the sulfate starved process phase (t^*). For all modeled linear fed-batch processes, right of the gray dashed line, no SO_4^{2-} limitation occurred. Panels **B** and **C** show the process curves of metabolites of interest in the optimal process. The gray dashed lines indicate the switching time point between the growth and production phases at $t = 22.3$ h instead of the initially predicted 23 h with constant q_{pDNA} (Figure 4).

A.3. Additional Notes

A.3.1. Optimal Sulfate Limited Batch Process

Methods Initial conditions (Table S1) represent a realistic batch process in a small bioreactor. Glucose uptake rate, $q_{\text{glucose}}(t)$, was modeled as Michaelis-Menten kinetics,

$$q_{\text{glucose}}(t) = \frac{q_{\text{glucose}}^{\text{max}} C_{\text{glucose}}(t)}{k_{\text{M}} + C_{\text{glucose}}(t)} \tag{S1}$$

with C_{glucose} denoting the current glucose concentration in the medium, and two constants ($q_{\text{glucose}}^{\text{max}}, k_{\text{M}}$). Simulations terminated once the glucose concentration dropped below the level of what was required for ATP maintenance. Initial sulfate concentration was varied in 301 equidistant steps to search for a volumetric pDNA productivity optimum.

Results We simulated sulfate limited batch processes using the initial values listed in Table S1 and analyzed the impact of the length of the pDNA production phase (induced by sulfate starvation) on the average volumetric productivity (Figure S1D).

We observe distinct maxima in the average volumetric productivity when $\kappa_{\text{pDNA}} > 1$ (red dotted line, Figure S1D), i.e. when the pDNA production rate is enhanced. With increasing κ_{pDNA}, the maximum productivity increases and moves towards smaller values of $C_{\text{SO}_4^{2-}}(0)$. The initial sulfate concentration (not shown) is proportional to the length of the growth phase. The larger $C_{\text{SO}_4^{2-}}(0)$, the longer the growth phase and the shorter the starvation (i.e., pDNA production) phase (t^*), and vice versa. Thus, Figure S1D mirrors the trade-off between producing a sufficiently high cell density that catalyzes the synthesis of pDNA, and actual pDNA production [42]. For large improvements, i.e., $\kappa_{\text{pDNA}} \gtrapprox 3$, the optimal length of the starvation phase is roughly 2.5 h and becomes virtually independent of κ_{pDNA}.

As an example, the optimal productivity for $\kappa_{\text{pDNA}} = 2$ (corresponding to $q_{\text{pDNA}}^* = 9.9\,\text{mg}\,\text{g}^{-1}\,\text{h}^{-1}$) is reached by adding $0.092\,\text{g}\,\text{L}^{-1}$ SO_4^{2-} into the batch medium. This results in a 1.7 h starvation phase, which increases the average volumetric productivity by 47 %. The time-dependent concentrations of this process are shown in Figure S1E and F.
